# Beyond brown: polyphenol oxidases as enzymes of plant specialized metabolism

**DOI:** 10.3389/fpls.2014.00783

**Published:** 2015-01-14

**Authors:** Michael L. Sullivan

**Affiliations:** U.S. Dairy Forage Research Center, Agricultural Research Service, United States Department of AgricultureMadison, WI, USA

**Keywords:** betalains, aurones, tyrosine, metabolism, L-DOPA, tyramine, 8-8’ linked lignans, specialized metabolism

## Abstract

Most cloned and/or characterized plant polyphenol oxidases (PPOs) have catechol oxidase activity (i.e., they oxidize *o*-diphenols to *o*-quinones) and are localized or predicted to be localized to plastids. As a class, they have broad substrate specificity and are associated with browning of produce and other plant materials. Because PPOs are often induced by wounding or pathogen attack, they are most generally believed to play important roles in plant defense responses. However, a few well-characterized PPOs appear to have very specific roles in the biosynthesis of specialized metabolites via both tyrosinase (monophenol oxidase) and catechol oxidase activities. Here we detail a few examples of these and explore the possibility that there may be many more “biosynthetic” PPOs.

Polyphenol oxidases (PPOs) are copper containing enzymes that are nearly ubiquitous among plants (Mayer, 2006). They have catechol oxidase activity (oxidation of *o*-diphenols to their corresponding *o*-quinones, EC 1.10.3.1) and many also have the ability to hydroxylate monophenols to *o*-diphenols (tyrosinase, EC 1.14.18.1; Marshall and Yoruk, 2003; Mayer, 2006). Throughout this review, the term tyrosinase will refer to the enzyme activity that oxidizes monophenols to *o*-diphenols. In the literature, the designation “PPO” sometimes includes laccases (EC 10.3.2). These copper containing enzymes are capable of oxidizing a wide range of aromatic compounds (including some utilized by PPO as defined above) and in plants are thought to have roles in radical coupling of monolignols to form lignin and flavanoid polymerization in the cell wall [see Mayer and Staples (2002) and the introduction of Turlapati et al. (2011) for reviews of laccase function in higher plants]. In the remainder of this review, laccases are not considered in the discussion of PPOs in plant specialized metabolism.

In plants, PPOs are perhaps best know for their role in post harvest browning: secondary reactions of PPO-generated *o*-quinones with cellular nucleophiles lead to the familiar discoloration of fresh produce and other plant materials (Vamos-Vigyazo, 1981). In some cases, these quinone reactions may be useful: for example in the so-called fermentation process in tea production (Subramanian et al., 1999) or in helping to preserve protein in forage crops (Lee et al., 2004; Sullivan and Hatfield, 2006). Generally, however, such browning reactions are thought of as a negative in food processing. Consequently, much research on PPO has been driven by this aspect of the enzyme. Because many PPOs are induced by wounding or pathogen attack, it has long been suggested that PPOs may play a role in defense responses. Indeed, it has been shown that PPO plays such a role in tomato (Thipyapong et al., 2004). Nonetheless, exactly what roles this nearly ubiquitous enzyme plays in normal plant growth and development are largely unknown. Because some PPOs have tyrosinase (hydroxylation of a monophenol to an *o*-diphenol) activity, it had long been suggested that PPO was responsible for the production of caffeic acid from *p*-coumaric acid (see for example Vaughan and Butt, 1969). It now seems likely that most plants actually use a cytochrome P450 enzyme for this conversion *in vivo* (Schoch et al., 2001; Franke et al., 2002). Still, the enzymatic properties of PPOs are potentially capable of providing important functions in plant specialized metabolism. Here we present a few cases where tyrosinase and/or catechol oxidase activities of specific PPOs have been proposed or demonstrated to have a crucial role in some aspect of plant specialized metabolism. Are these cases exceptional, or the tip of the iceberg?

## BETALAIN BIOSYNTHESIS

**FIGURE 1:**
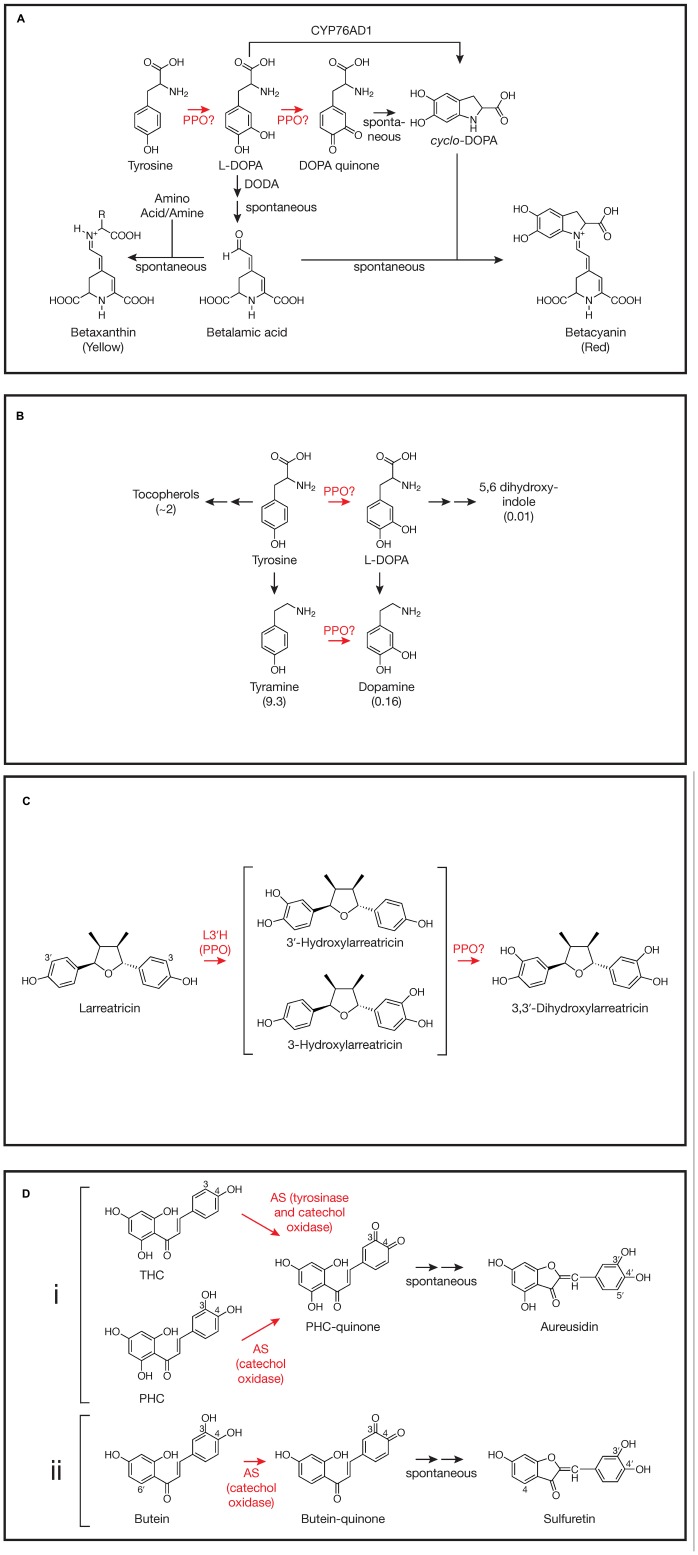
**Involvement of polyphenol oxidases (PPOs) in plant specialized metabolism.** Steps where PPO involvement has been demonstrated or proposed are highlighted in red. For simplicity, not all reactants, enzymes, or stereochemistry are shown. Steps that occur spontaneously (not mediated by an enzyme) are indicated. **(A)** Betalain synthesis as described by Hatlestad et al. (2012), Gandia-Herrero and Garcia-Carmona (2013), and others. DODA, DOPA 4,5-dioxygenase; CYP76AD1, the cytochrome P450 described by Hatlestad et al. (2012). The reaction mediated by CYP76AD1 likely proceeds via a DOPA quinone intermediate (Gandia-Herrero and Garcia-Carmona, 2013). **(B)** Proposed tyrosine metabolism in walnut (Araji et al., 2014). Values in parentheses are fold change in metabolite in walnut plants with PPO silenced via RNAi relative to wild type control plants as reported by Araji et al. (2014). Tyrosine levels did not change and L-DOPA itself was not detectable. **(C)** 3,3′-Dihydroxylarreatricin biosynthesis in creosote bush as proposed by Cho et al. (2003). L3′H, larreatricin 3′-hydroxylase. **(D)** (i) Aureusidin biosynthesis in *A. majus* as proposed by Nakayama et al. (2001). AS, aureusidin synthase (here, a vacuolar PPO); THC, 2′,4′,6′,4-tetrahydroxychalcone; PHC, 2′,4′,6′,3,4-pentahydroxychalcone. For simplicity, bracteatin formation from PHC by AS is not shown. (ii) Sulfuretin biosynthesis in *C. grandiflora* as proposed by Kaintz et al. (2014). AS, aurone synthase (here, a plastidic PPO).

There are several steps in betalain biosynthesis that might utilize either the tyrosinase and catechol oxidase activities of PPO (see Gandia-Herrero and Garcia-Carmona, 2013 for a detailed review). The first step in betalain biosynthesis is conversion of tyrosine to L-DOPA (L-3,4-dihydroxyphenylalanine; **Figure 1A**). The resulting L-DOPA can be a substrate for DOPA 4,5-dioxygenase (DODA) that cleaves DOPA’s aromatic ring to form 4,5-seco-DOPA. The cleavage product spontaneously rearranges to form betalamic acid, which can condense with amino acids or other amine groups to form yellow betaxanthins. Condensation of betalamic acid with *cyclo*-DOPA forms the red betacyanin pigments. The catechol oxidase activity of PPO could be involved in the oxidation of DOPA to DOPA quinone that can spontaneously rearrange to form the *cyclo*-DOPA moiety of the red betacyanin betalains (see Gandia-Herrero and Garcia-Carmona, 2013 and references therein). However, recently a cytochrome P450, CYP76AD1, has been identified in beet via a bioinformatic approach that appears to carry out this reaction *in vivo* and can compliment the R mutant (produces yellow, but not red pigment) in beet and other species (Hatlestad et al., 2012). It has been suggested that CYP76AD1 could also be responsible for the initial tyrosine to L-DOPA oxidation as well. If this is the case in beet, however, the activity would be redundant with another, since silencing of CYP76AD1 results in loss of red, but not yellow pigments whose formation do not require *cyclo*-DOPA formation (Hatlestad et al., 2012). For the most part, the tyrosinase activity of PPO has been presumed to be the enzyme that mediates the initial tyrosine to L-DOPA conversion. Many studies show a correlation between tyrosinase enzyme activity and/or PPO gene expression and betalain pigment formation (see Steiner et al., 1999; Chang-Quan et al., 2007; Gao et al., 2009, for example). Further, a betaxanthin pathway can be recreated in tobacco cells using fungal PPO to carry out the tyrosine to L-DOPA step (Nakatsuka et al., 2013). However, transcriptome analysis in beet did not find the abundance of PPO transcripts that might be expected for high betalain production (Hatlestad et al., 2012), nor to our knowledge have PPO gene silencing experiments demonstrated a role for PPO *in vivo*. Thus, despite longstanding speculation that PPO is involved in betalain biosynthesis, its role in *cyclo*-DOPA formation seems unlikely, and definitive demonstration of a role in the initial conversion of tyrosine to L-DOPA *in vivo* is lacking.

## TYROSINE METABOLISM IN WALNUT

Although a role for PPO in L-DOPA formation in betalain biosynthesis is far from clear, work by Araji et al. (2014) in walnut does support PPO-mediated conversion of tyrosine to L-DOPA, at least in some species. In walnut (*Juglans regia*), PPO is encoded by a single gene and has been demonstrated to have both tyrosinase and catechol oxidase activity (Escobar et al., 2008). To examine the *in vivo* function of PPO in walnut, Araji et al. (2014) created several RNAi transgenic lines that showed >95% reductions in catechol oxidase activity relative to wild type controls. When placed in soil, these plants had a striking phenotype: they developed disease-like necrotic lesions. Despite the lesions, no pathogens could be identified from the leaves. Levels of salicylic acid, H_2_O_2_, or malondialdehyde (an indicator of oxidative damage), previously associated with other lesion-mimic mutants (Lorrain et al., 2003), were not significantly different in the PPO-silenced leaves compared to those of wild type leaves. Metabolomic analysis of PPO-silenced and wild type leaves did reveal significant differences in many metabolites, however, particularly phenylpropanoids. Especially striking were changes in levels of compounds associated with tyrosine metabolism (**Figure 1B**; Araji et al., 2014). Compared to wild type leaves, those from PPO-silenced plants, had massively increased levels of tyramine (nearly 10-fold), a primary metabolite of tyrosine, and substantial increases in tocopherols (∼twofold), secondary metabolites of tyrosine. Conversely, levels of metabolites that would be expected to be derived from the 3-hydroxylation of tyrosine or tyramine (both good substrates for the tyrosinase activity of walnut PPO *in vitro*) were markedly reduced in PPO-silenced plants. Although L-DOPA was undetectable in both PPO-silenced and wild type walnut plants, levels of dopamine (derived from either L-DOPA or tyramine) and 5,6 dihydroxyindole (derived from L-DOPA) were reduced approximately 6- and 100-fold, respectively, in PPO-silenced plants relative to wild type controls (**Figure 1B**). Because the enzyme involved in 3-hydroxylation of these compounds had not been previously identified, the authors proposed that the simplest interpretation of the metabolomic results is that walnut PPO is the enzyme that mediates 3-hydroxylation of tyrosine and tyramine (Araji et al., 2014). Thus, silencing of PPO would be expected to result in increased accumulation of those tyrosine metabolites that do not undergo 3-hydroxylation such as tyramine and the tocopherols and decreased accumulation of metabolites derived from L-DOPA or tyramine. Further, the authors were able to demonstrate that the necrotic lesion phenotype of the PPO-silenced plants was almost certainly due to the accumulation of tyramine: incubation of petioles of detached wild type leaves in tyramine solution could phenocopy the necrotic lesions (Araji et al., 2014). Another metabolite that was dramatically decreased in PPO-silenced plants was esculetin. Although biosynthesis of this compound is not well understood, this observation is consistent with previous suggestion of the involvement of a chloroplast localized phenolase (Sato, 1967). More definitive demonstration of a central role of walnut PPO in tyrosine metabolism and esculetin biosynthesis in walnut might require approaches such as radioactive pulse labeling. It will be interesting to see how widespread this role of PPO in tyrosine metabolism is, especially in species whose PPO enzymes have been shown to have tyrosinase activity.

## BIOSYNTHESIS OF 8-8’ LINKED LIGNANS IN CREOSOTE BUSH

Another likely case of the tyrosinase activity of a PPO being involved in biosynthesis of a specialized metabolite is in the formation of 8-8′ linked lignans in creosote bush (*Larrea tridentata*). Many of these compounds from creosote bush, e.g., nordihydroguaiaretic acid (NDGA), have a number of bioactive properties including antiviral (Craigo et al., 2000), anticancer (McDonald et al., 2001) and allelopathtic properties (Elakovich and Stevens, 1985). Cho et al. (2003) used a combination of radiolabeled precursor experiments and metabolite identification to investigate the pathway of formation of NDGA and related compounds. Focusing on one of the steps, they postulated aromatic ring hydroxylation of larreatricin to form 3′ or 3-dihydroxylarreatricin (**Figure 1C**). A protein preparation from creosote bush was found to be capable of this activity, forming the 3′- and 3-hydroxylarreatricin compounds in a ratio that favored the 3′ compound by ∼seven fold. The larreatricin 3′-hydroxylase activity was purified to apparent homogeneity and showed enantiospecific hydroxylation, converting (+)-larreatricin to the corresponding (+)-3′-hydroxylarreatricin, but not the (-)-enatomer. Peptide sequencing of the purified hydroxylase identified fragments with high homology to conserved domains of PPOs from other plant species. The peptide sequence data further allowed cloning and sequencing of a full length cDNA corresponding to the *L. tridentata* PPO. Like most plant PPOs, the *L. tridentata* PPO contains N-terminal sequences that would predict its localization to the chloroplast thylakoid lumen suggesting at least some of the steps of the synthesis of NDGA and related compounds take place in plastids. Unfortunately, in this study, reverse genetics (e.g., a gene silencing experiment) was not (or could not) be done nor was a recombinant protein product (e.g., produced in *Arabidopsis*, which lacks an endogenous PPO) characterized to more conclusively demonstrate the *L. tridentate* PPO gene product acts as the (+)-larreatricin 3′-hydroxylase *in vivo*. Nonetheless, the apparent homogeneity of the (+)-larreatricin 3′-hydroxylase purified from *L. tridentate* and its enantio-specificity for (+)-larreatricin are compelling. Unfortunately, there appears to have been relatively little further work on this pathway or characterization of the *L. tridentate* PPO. It would be interesting to know, for example, whether this same PPO mediates a second hydroxylation to form 3,3′-dihydroxylarreatricin (**Figure 1C**), and if not, what enzyme carries out that reaction. Does this PPO also form quinones from hydroxylarreatricins and under what conditions? Would such activities have any biological implications?

## AURONE BIOSYNTHESIS

One of the most interesting and well-studied cases of PPO having a role in biosynthesis of specialized metabolites is the biosynthesis of the chalcone-derived yellow aurone pigments in snapdragon (*Antirrhinum majus*) flowers. It had been found that aurones (aureusidin and bracteatin) were formed from 2′,4′,6′,4-tetrahydroxychalcone (THC) or 2′,4′,6′,3,4-pentahydroxychalcone (PHC) upon incubation with extracts of yellow snapdragon flowers (Sato et al., 2001). The enzyme responsible, aureusidin (or aurone) synthase (AS), was purified to homogeneity from yellow snapdragon buds (Nakayama et al., 2000). Peptide sequencing of the purified enzyme allowed isolation and characterization of a cDNA encoding the enzyme. The predicted protein sequence showed high homology to other plant PPO enzymes. Expression of the gene corresponded to aurone accumulation (e.g., it was expressed in yellow flowers, but not in white or red flowers, nor in leaves) and expression developmentally coincided with levels of AS activity. Further, *in vitro*, tyrosinase from *Neurospora crassa* could also convert THC to aureusidin, indicating that the enzymatic activities of PPO are involved in the biosynthetic conversion. Subsequent detailed studies of AS substrate specificity allowed elucidation of a likely mechanism of aurone formation from THC or PHC involving both tyrosinase and catechol oxidase activities of the AS PPO (**Figure 1Di;** Nakayama et al., 2001). Starting with THC, tyrosinase and catechol oxidase activity result in 3-hydroxylation and formation of the corresponding *o*-quinone. Whether AS PPO carries out the 3-hydroxylation reaction *in vivo*, or whether a cytochrome P450 chalcone 3-hydroxylase (as described below for *Coreopsis grandiflora*) is also involved has not been definitively established. AS likely forms the same quinone from PHC without the need for the 3-hydroxylation step. The resulting quinone is predicted to undergo a 2-step non-enzyme mediated rearrangement to form aureusidin (Nakayama et al., 2001). Although the major product formed from PHC by AS is the 3′,4′-hydroxylated aureusidin, smaller amounts (approximately 1/6 as much) of the 3′,4′,5′-hydroxylated bracteatin are also formed (not shown in **Figure 1Di**), suggesting AS is capable of adding a 5-hydroxyl to the PHC substrate. The 5′-hydroxylation of aureusidin by AS can be ruled out as the mechanism of bracteatin formation since incubation of aureusidin with AS failed to produce the product. AS was also unable to oxidize aureusidin to its corresponding quinone, nor could it oxidize several other mono and *o*-diphenolic compounds, such as tyrosine, *p*-coumaric acid, L-DOPA, caffeic acid, or eriodictyol, suggesting a relatively strict substrate specificity (Nakayama et al., 2001). One of the most novel aspects of the *A. majus* AS PPO is that it lacks the usual chloroplast targeting information that is common to most characterized PPOs (Nakayama et al., 2000). Subsequent studies using density gradient fractionation and GFP-fusions with AS sequences demonstrated that AS is localized to vacuoles, where 4′-glucosides of the chalcone substrates, which may be the native substrates for AS, are also localized (Ono et al., 2006b). Consistent with this, Ono et al. (2006a) were able to produce aurones in flowers normally lacking them by expressing both the *A. majus* AS gene and a gene encoding chalcone 4′-*O*-glucosyltransferase from *A. majus*. Both genes were required, leading to the conclusion that chalcones are 4′-glycosylated in the cytoplasm leading to their transport to the vacuole where they can serve as substrates for *A. majus* AS. Recently, Kaintz et al. (2014) identified a PPO from *C. grandiflora* whose expression pattern is consistent with it being responsible for 4-deoxyaurone formation in this species (**Figure 1Dii**). Interestingly, the *C. grandiflora* AS PPO has relatively low sequence identity with *A. majus* AS PPO, is predicted to be plastid localized, and appears to lack tyrosinase activity. Consistent with the lack of tyrosinase activity, the *C. grandiflora* AS cannot utilize 4-monophenolic chalcone substrates and it seems likely that a cytochrome P450 chalcone 3-hydroxylase produces the 3,4-dihydroxy chalcones utilized by this AS (Schlangen et al., 2010; Kaintz et al., 2014). The plastidic versus vacuolar nature of the *C. grandiflora* and *A. majus* AS enzymes, respectively, indicate differences in the cell biology of aurone formation in these two systems, despite sharing some underlying PPO-mediated-chemistry.

## CONCLUDING REMARKS

The above examples could represent the tip of the iceberg with respect to PPO enzymes that have specific roles in biosynthesis of specialized metabolites. Much work on PPOs has focused on their negative impact on food quality due to the browning reactions they promote. It could be that most of these characterized “food quality” PPOs are involved in general defense responses, leaving the impression that most PPOs are not particularly specialized. In two of the cases above, the specialized roles of the PPOs were identified in the course of research focused on a particular aspect of specialized metabolism. There, relatively laborious approaches led to the identification of the PPOs involved. For example, for both larreatricin 3′-hydroxylase (Cho et al., 2003) and *A. majus* AS (Nakayama et al., 2000), multistep protein purifications utilizing large amounts of plant tissue identified these PPO enzymes (for AS, 90 μg of enzyme was purified from 32 kg of snapdragon buds!). Bioinformatics approaches will almost certainly facilitate identifying these specialized PPOs in the future. For example, in an analysis of PPO gene families from land plants whose genomes had been sequenced, Tran et al. (2012) identified 17 out of 83 PPO genes which lacked a chloroplast targeting signal and instead had either secretory pathway or unknown intracellular targeting. Although some of these could certainly be involved in defensive responses, such as seed defense (Anderson et al., 2010; Fuerst et al., 2014), it is intriguing to think that some represent PPOs involved in very specific processes including specialized metabolism, like the vacuolar targeted *A. majus* AS (Ono et al., 2006b). As they are becoming more routine, transcriptomic, metabolomic, and proteomic analyses could also provide useful information related to PPO function as was the case for walnut PPO discussed above. These types of analyses can provide answers to questions such as whether expression of a given PPO is tightly correlated with a phenotype of interest or whether a particular PPO is present in the subcellular compartment where a specific biosynthetic reaction is thought to occur. Thus genomics data, combined with other bioinformatic approaches, will almost certainly facilitate better understanding of PPO function in general, and the roles specific PPOs may play in specialized metabolism.

## Conflict of Interest Statement

The author declares that the research was conducted in the absence of any commercial or financial relationships that could be construed as a potential conflict of interest. The Guest Associate Editor Dr. K. Judith Webb declares that, despite having collaborated with author Dr. Michael L. Sullivan, the review process was handled objectively and no conflict of interest exists.
